# Comparison of Coping Strategies and Resilience Among White, Non-White, and Asian Older Adults in San Francisco

**DOI:** 10.1093/geroni/igaa057.1929

**Published:** 2020-12-16

**Authors:** Rashmi Gupta, Vijayan Pillai

**Affiliations:** 1 San Francisco State University, Daly City, California, United States; 2 University of Texas at Arlington, Arlington, Texas, United States

## Abstract

There has been a substantial increase in research on coping strategies/resilience in later life in the last decade (Moos, et al., 2006). However studies exploring the differences in coping strategies/resilience in later life among various ethnic groups such as Whites, non-Whites and Asians are few. The purpose of this study is to explore the similarities and differences in coping strategies/resilience among a diverse group of 30 older adults age 65 and above. A purposive sample of older adults was recruited from a local senior center and fitness program in the West coast. A questionnaire was developed with open ended questions. We conducted face to face interviews with seniors. Results indicated that there were similarities in terms of coping/resilience strategies among various ethnic groups in that they relied primarily on friends for support. The sample respondents were living in their own homes and were physically active, despite health issues.

